# Hierarchical Organization of Auditory and Motor Representations in Speech Perception: Evidence from Searchlight Similarity Analysis

**DOI:** 10.1093/cercor/bhv136

**Published:** 2015-07-08

**Authors:** Samuel Evans, Matthew H. Davis

**Affiliations:** 1 MRC Cognition and Brain Sciences Unit, CambridgeCB2 7EF, UK; 2 Institute of Cognitive Neuroscience, University College London, WC1 3AR, UK

**Keywords:** fMRI, hierarchical processing, multivoxel pattern analysis, representational similarity analysis, speech perception

## Abstract

How humans extract the identity of speech sounds from highly variable acoustic signals remains unclear. Here, we use searchlight representational similarity analysis (RSA) to localize and characterize neural representations of syllables at different levels of the hierarchically organized temporo-frontal pathways for speech perception. We asked participants to listen to spoken syllables that differed considerably in their surface acoustic form by changing speaker and degrading surface acoustics using noise-vocoding and sine wave synthesis while we recorded neural responses with functional magnetic resonance imaging. We found evidence for a graded hierarchy of abstraction across the brain. At the peak of the hierarchy, neural representations in somatomotor cortex encoded syllable identity but not surface acoustic form, at the base of the hierarchy, primary auditory cortex showed the reverse. In contrast, bilateral temporal cortex exhibited an intermediate response, encoding both syllable identity and the surface acoustic form of speech. Regions of somatomotor cortex associated with encoding syllable identity in perception were also engaged when producing the same syllables in a separate session. These findings are consistent with a hierarchical account of how variable acoustic signals are transformed into abstract representations of the identity of speech sounds.

## Introduction

How do listeners perceive highly variable speech signals? No two naturally produced syllables are exactly alike since their precise acoustic realization varies both within and between speakers. Despite this variability, listeners are typically able to understand speech rapidly and accurately even when it has been significantly degraded (Remez et al. 1981; Shannon et al. 1995; Dupoux and Green 1997; Brungart 2001). These observations suggest that no single acoustic cue is necessary for correct perception of speech sounds. Historically, the absence of reliable acoustic cues to speech sound identification led researchers to suggest that speech is understood by reference to the intended motor gestures of the speaker (Studdert-Kennedy et al. 1970; Liberman and Whalen 2000). Recent demonstrations that motor cortex responds to clear and degraded speech (Wilson et al. 2004; Pulvermüller et al. 2006; Hervais-Adelman et al. 2012) and that transcranial magnetic stimulation (TMS) of motor regions disrupts speech perception (Meister et al. 2007; Möttönen and Watkins 2009) are consistent with a functional role of motor cortex in perception. Yet, the nature of motor contributions to speech perception remains controversial (Lotto et al. 2009; Scott et al. 2009), and the neural mechanisms by which motor representations are accessed from speech remain underspecified.

Here, we examine the representational content of neural responses to speech in auditory and motor cortex. We test the proposal that motor regions sit at the peak of the hierarchically organized temporo-frontal processing pathways that respond to speech. We show that motor regions represent speech in a way that is more fully abstracted from the surface acoustic form than representations in auditory regions. We situate these observations in the context of two long-standing debates in the cortical organization of speech processing: 1) the role of motor representations in speech perception and 2) the hierarchical organization of auditory and speech pathways.

### Motor Representations of Heard Speech

Functional magnetic resonance imaging (fMRI) and TMS studies are consistent with a role for motor cortex in the perception of clear and degraded speech (Wilson et al. 2004; Pulvermüller et al. 2006; Meister et al. 2007; D'Ausilio et al. 2009; Möttönen and Watkins 2009). However, these findings are controversial for methodological reasons (e.g., the lack of subtractions from non-speech baselines [Scott et al. (2009)]) and arguments that activity only arises from working memory and other task demands that are not specific to speech (Lotto et al. 2009; McGettigan et al. 2010). Indeed similar findings are not always obtained in more natural speech comprehension tasks (Krieger-Redwood et al. 2013) or when the response to speech is compared with acoustically complex non-speech baselines (Scott et al. 2000, 2006). Thus, it is still argued that motor cortex activity is not necessary for everyday speech perception (Hickok et al. 2011).

However, there is fMRI evidence for motor cortex recruitment during comprehension of degraded speech, when compared with clear speech, even when simple auditory detection tasks are used (Osnes et al. 2011; Hervais-Adelman et al. 2012). These findings argue against the proposal that motor responses are only observed during tasks that require maintenance of speech sounds in working memory or categorical judgments of segment identity. However, the nature of the representations that are activated in motor regions remains unclear, and overlap with regions activated during speech production has not been unambiguously shown. Activation in more inferior regions of frontal cortex has also been shown in response to both sine wave (SW) (Dehaene et al. 2005) and noise-vocoded speech (Eisner et al. 2010) and has often been attributed to working memory, control, and decision processes (Binder et al. 2004; Myers et al. 2009; Eisner et al. 2010). It is possible that existing demonstrations of motor activity can be explained as arising from similar processes. While functional imaging data cannot provide causal evidence that motor activity is necessary for speech perception, the spatial resolution of fMRI combined with multivariate analysis methods allows us to assess the nature of the information that is encoded within neural regions. Here, we use these methods to assess whether motor regions activated during speech production represent the identity of clear and degraded syllables during a simple repetition detection task.

### Hierarchical Organization of Auditory and Speech Pathways

Existing studies provide evidence for a graded hierarchy of responses to sounds in superior and lateral regions of the temporal lobe. Hierarchical organization of auditory fields in primates is well established in anatomical and electrophysiological studies (Rauschecker 1998; Kaas and Hackett 2000). Similar hierarchical organization has been suggested in the context of human functional imaging. Primary auditory cortex (PAC) responds to pure tones, whereas surrounding auditory regions in lateral Heschl's gyrus and planum temporale respond to more complex band-passed noises (Binder et al. 2000; Wessinger et al. 2001).

Within this hierarchy, speech-specific responses to isolated syllables are only observed in later stages of processing, primarily in the superior temporal sulcus (Liebenthal et al. 2005; Uppenkamp et al. 2006; Heinrich et al. 2008). While some of these findings might reflect acoustic differences between speech and non-speech stimuli, studies with SW speech have demonstrated that acoustically identical stimuli evoke additional responses in the posterior STS when they are perceived as speech (Dehaene et al. 2005; Möttönen et al. 2006; Desai et al. 2008). Similar regions of posterior STS (and adjacent inferior parietal cortex) are activated for categorical perception of syllables, for example, showing an additional response to sequences that include categorical changes, compared with repetition or within-category changes (Jacquemot et al. 2003; Zevin and McCandliss 2004; Joanisse et al. 2007; Raizada and Poldrack 2007). However, studies with non-speech analogs have also demonstrated additional activity in these regions when listeners are trained to perceive non-speech sounds categorically (Leech et al. 2009). Thus, it might be that posterior STS regions are activated for any kind of categorically perceived sound rather than representing speech content *per se*.

Multivariate pattern analysis (MVPA) methods have a unique role to play in establishing the nature of the neural representations in auditory and lateral temporal regions (Haynes and Rees 2006; Kriegeskorte et al. 2008; Mur et al. 2009). MVPA analysis of fMRI responses to speech has shown that neural responses in both anterior and posterior STS can distinguish intelligible and unintelligible sentences irrespective of their acoustic form (Okada et al. 2010; Evans, Kyong et al. 2014). This profile suggests a functional distinction between anterior and posterior temporal cortex and regions closer to PAC which distinguish stimuli that differ in their surface acoustic structure better than they distinguish stimuli which differ in intelligibility (Okada et al. 2010). However, these studies examined responses to sentence length materials that were either intelligible or unintelligible. Hence, differential responses may be associated with lexical and syntactic processing of speech as well as lower level perceptual processes.

In order to focus on purely perceptual processes, studies of speech processing using MVPA have assessed the categorical content of isolated syllables rather than sentences (Formisano et al. 2008; Kilian-Hutten et al. 2011). One key method for demonstrating abstract, non-acoustic representations of speech is to show that decoding of multivariate patterns generalizes across different acoustic tokens (e.g., from one speaker to another). These studies have typically shown abstract encoding of syllable identity in peri-auditory areas of the superior temporal gyrus (STG) (Formisano et al. 2008; Chang et al. 2010; Kilian-Hutten et al. 2011). These findings might suggest that abstract representations of speech sounds can also be found in early auditory regions, thereby challenging hierarchical accounts. However, since these studies did not examine responses outside the temporal lobe or compare encoding of acoustic vs. abstract speech representations, the results do not speak to hierarchical organization of speech perception networks in lateral temporal regions. Recent MVPA studies have shown that neural responses in inferior frontal and motor regions discriminate ambiguous (Lee et al. 2012) or noisy syllables (Du et al. 2014). However, in these studies, the degree of abstraction demonstrated remains unclear.

Here, we used whole-brain searchlight representational similarity analysis (RSA) (Kriegeskorte et al. 2006, 2008) to assess neural coding of isolated syllables. Participants heard 1 of 6 syllables, spoken by 2 speakers and presented in 3 different acoustic forms. Using these methods, we can assess whether neural representations in auditory, lateral temporal, and motor regions code for the surface acoustic form or underlying identity of isolated syllables. In this way, we can test for acoustic sensitivity at different levels of the speech processing hierarchy, with the goal of localizing the stages by which variable acoustic signals are transformed into more abstract representations of syllable identity. Furthermore, by assessing similarity between pairs of non-identical syllables that share phonetic features, phonemic units or syllabic structure, we can also assess the nature of syllable representations at different processing stages.

## Method

### Participants

Eighteen right-handed native speakers of British English aged between 18 and 40 (mean age: 27, 6 males) were scanned. All participants reported being without any hearing or language impairment and gave informed consent to take part in the study that was approved by the Cambridge Psychology Research Ethics Committee. Participants were paid a nominal fee to participate. One participants' fMRI data were shown to contain a “ghosting” artifact and was removed from analysis. The final data set included 17 participants.

### Stimuli

Six speech tokens: /ba/, /da/, /ma/, /na/, /ab/, /ad/, were recorded using 16-bit quantization and a 44.1-kHz sampling rate, in a sound-isolated booth by a male and a female speaker of southern British English. The syllables were of a similar duration (mean = 550 ms, min = 485 ms, max = 612 ms). They were produced in isolation, for example, not in the context of a longer spoken utterance, explaining their relatively long duration. The natural speech tokens were further processed to generate 3 conditions: clear (CL), 3 formant SW, and noise-vocoded (NV) speech. This generated 36 unique stimuli: 3 acoustic forms (CL, SW, NV) × 2 speakers (male/female) × 6 syllable identities (/ba/, /da/, /ma/, /na/, /ab/, /ad/). Both consonant vowel (CV) and VC contexts were included to increase acoustic variability.

Noise vocoding was conducted using the technique described by Shannon et al. (1995) using custom scripts written in Matlab. The syllables were filtered into 16 logarithmically spaced frequency bands from 94 to 4525 Hz (Greenwood 1990) with each pass band 3 dB down with a 16 dB/octave roll off. In each band, amplitude envelopes were extracted using half wave rectification, and pitch synchronous oscillations above 30 Hz were removed with a second-order Butterworth filter. The resulting envelopes were multiplied with a broadband noise, band-pass-filtered in the same frequency ranges as the source, and recombined.

Three-formant SW speech (Remez et al. 1981) was created using linear predictive coding (LPC) to estimate the frequency and amplitude of SWs tracking the formants of speech. This was achieved using Matlab scripts written by Dan Ellis, available at: http://www.ee.columbia.edu/ln/labrosa/matlab/sws/, last accessed June 15, 2015. Previous work has shown that hand correction of formant tracks significantly improves the intelligibility of SW speech (Remez et al. 2011). Hence, the original frequency-amplitude estimates derived from the LPC analysis and smoothed estimates, in which high-frequency noise had been removed, were overlaid on a spectrogram to guide hand correction. Formant frequency and amplitude estimates were hand-corrected using custom Matlab scripts that allowed values to be edited using an interactive display. The stimuli were then resynthesized. Note that SW resynthesis removes pitch cues that would aid speaker identification and voice-based gender judgments; it does however preserve other aspects of speaker information such as formant dispersion (e.g., the average spacing between formants).

**Figure 1.**
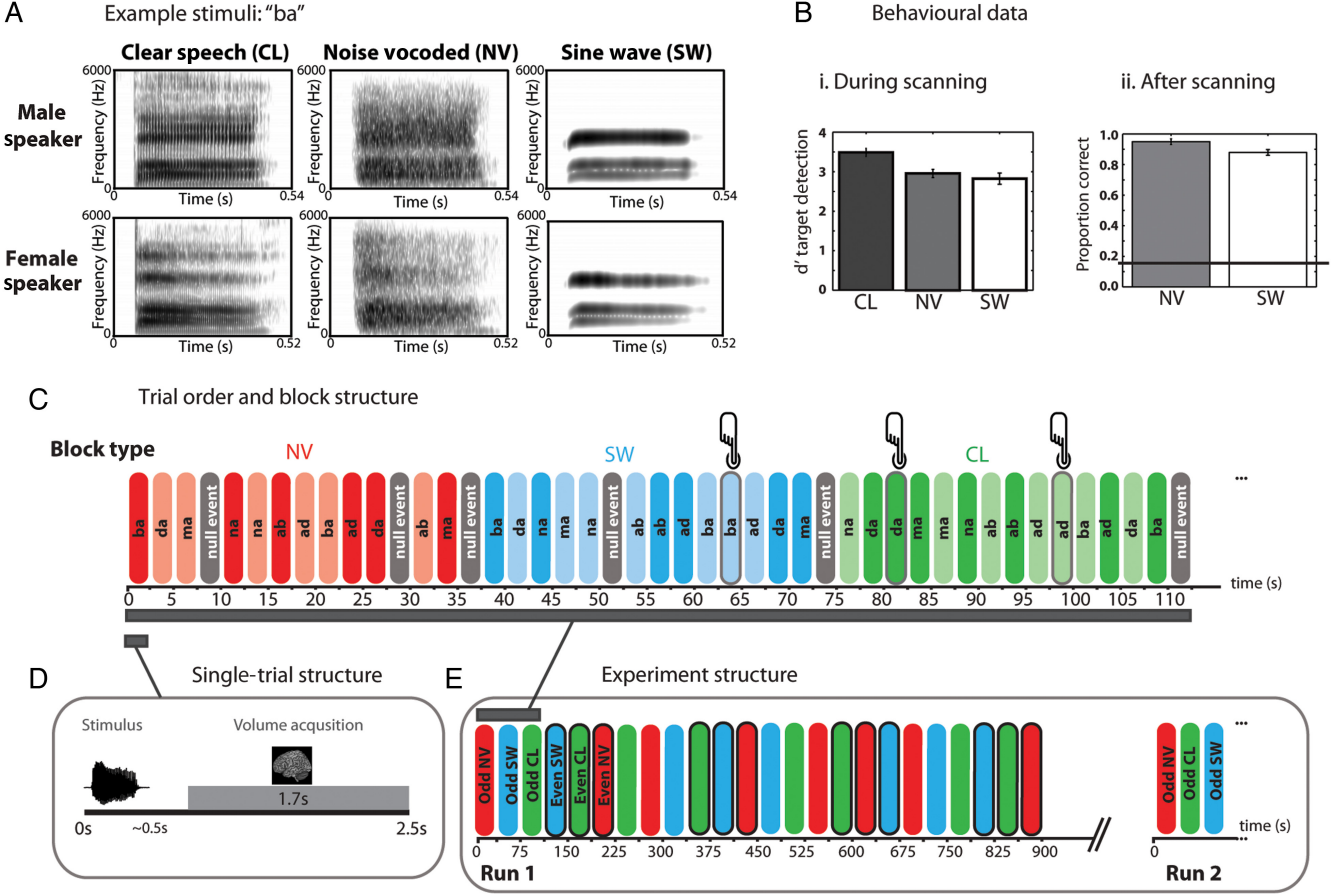
(*A*) Spectrograms of /ba/ syllables presented as clear speech (CL), NV speech (NV), and SW resynthesized (SW). (*B*) Behavioral data i) one-back repetition detection accuracy during scanning and ii) proportion correct identification of degraded syllables after scanning (horizontal line shows chance level of 0.16). (*C*) Block structure showing trial sequences during an example scanning run. Red = NV syllables, blue = SW syllables, green = CL syllables, grey = null trials. Dark coloring = male speaker, light coloring = female speaker. Grey outer shading plus button icon indicates repetition trials. (*D*) Single-trial structure showing the timing of stimulus presentation and MR volume acquisition. (*E*) Experiment structure showing odd- and even-numbered experimental blocks in each scanning run.

Clear speech tokens were low-pass-filtered at 6 kHz to mimic the band limit applied by the noise-vocoding routine and to equate the distribution of spectral energy with the SW speech, which lacked spectral energy of >6 kHz. All stimuli were then root-mean-square-equalized to equate their intensity. Please refer to Figure 1*A* to see example spectrograms of the stimuli.

### PreScanning Training

Each participant was trained to understand the NV and SW speech in the week prior to the fMRI scanning session. The aim of this training was to make the degraded speech conditions as intelligible as possible prior to scanning (at least 85% correct report for both conditions). The training session varied in duration depending on the performance of each participant, for most participants, it lasted between 60 and 90 min. Two participants required further training to reach high levels of accuracy and were therefore asked to return on a different day for a further training session lasting an additional 30 min.

Training was conducted separately for NV and SW speech. The order in which these conditions were trained was counterbalanced across participants. Training was presented in blocks lasting ∼3 min. In the first block of each degradation type, the participant heard each degraded syllable from each speaker once (12 trials in total), then in subsequent blocks, each token was presented twice (24 trials in total) in random order. After each stimulus was presented, participants were required to perform a 6-alternative forced-choice syllable identification task by using a mouse to click a button on which the identity of each syllable was written. Participants were allowed to hear the degraded speech token again if they wished before making their response. After every response, the participants received visual feedback and heard the syllable in its clear and then its degraded form (a procedure previously shown to enhance learning of degraded speech [Davis et al. 2005]). After each block, participants were given feedback on their overall accuracy in the preceding trials and shown the syllable confusions that they had made. They were then presented with both the clear and degraded versions of the syllables they had confused and were given an opportunity to replay any other additional sounds they wished. This process continued until each participant scored >90% (22 of 24 trials correct) on 3 consecutive blocks or until 45 min of training had elapsed (in the instance that the previous condition had not been met). They were then tested with 10 repetitions of each syllable (120 trials in total), without corrective feedback, to obtain a final measure of their performance after training. If their accuracy for either degradation type was shown to be <85% (<21/24 correct), the participant was asked to return for an additional training session.

### Scanning

On the day of scanning, each participant was re-familiarized with the SW and NV syllables using the same training procedure described in the section above. The fMRI scanning session comprised a speech production task (10 min), a speech perception task (4 scanning runs totaling 60 min), and a structural scan (5 min), presented in that order. Prior to scanning, participants were briefed on the tasks that they were required to perform in the scanner and completed practice trials. After the end of the scanning session, each participant's perception of the degraded syllables was tested again using the testing procedure described previously (except for one participant who was unable to complete testing due to time constraints).

MRI data were acquired on a 3-Tesla Siemens Tim Trio scanner using a 32-channel head coil. A T1-weighted structural scan was acquired for each subject using a three-dimensional MPRAGE sequence (TR: 2250 ms, TE: 2.99 ms, flip angle: 98°, field of view: 256 × 240 × 160 mm, matrix size: 256 × 240 × 160 mm, spatial resolution, 1 × 1 × 1 mm).

### Speech Production Task

For each participant, 281 echo planar imaging (EPI) volumes comprising thirty-two 3-mm-thick slices were acquired using a continuous, descending acquisition sequence (TR = 2000 ms, TA = 2000 ms, TE = 30 ms, FA = 78°, matrix size: 64 × 64, in-plane resolution: 3 × 3 mm, interslice gap = 25%). We used transverse-oblique acquisition, with slices angled away from the eyeballs to avoid ghosting artifacts from eye movements. The FOV was chosen to include as much of the frontal and temporal cortex as possible, at the expense of superior parietal cortex. All experimental tasks within the scanner were presented using E-Prime. Participants were asked to read aloud (“Loud”) or covertly (“Self”) in blocks of trials lasting 18 s in which all 6 syllables were produced twice in a random order. These 2 conditions alternated with a short period of silent rest. The scanning run contained 10 repetitions of each active block and 20 rest blocks. In these “active” trials, the instruction, “Loud” or “Self”, above a central fixation cross was presented on a screen for 0.5 s. The fixation cross was then replaced by a written syllable that was presented for a further 1 s. Each syllable was repeated twice in a randomized order in each block. The rest blocks lasted for 9 s, during which time the participants saw the word “Rest” with a fixation cross below it. Participants spoke into a microphone attached to the head coil. The audio of each session was monitored to ensure that participants engaged appropriately in the task.

### Speech Perception Task

For each participant, 1464 EPI volumes, each comprising 26 × 3 mm-thick slices, were acquired with a fast sparse protocol such that speech stimuli could be presented in the 800-ms silent gap between volume acquisitions (TR = 2500 ms, TA = 1700 ms, TE = 30 ms, FA = 78°, matrix size: 64 × 64, in-plane resolution: 3 × 3 mm, slice gap = 25%). The FOV used in the speech production task was copied over to the speech perception task, but centered on the middle slice such that whole-brain coverage was achieved except for the most superior portion of the parietal lobe and anterior, inferior portions of the temporal lobe. On each trial, a single syllable was played in the 800-ms silent interval between volume acquisitions (Fig. 1*D*). During the scanning session, participants performed a one-back detection task to ensure attention to the speech stimuli. Participants pressed a button held in their left hand whenever they heard 2 consecutive presentations of the same syllable in the same acoustic form (e.g., 2 consecutive NV /ba/ syllables produced by a male speaker). The randomization of the syllables was constrained so that for repetition trials, consecutive syllables spoken by different speakers in the same acoustic form were not presented, to ensure that participants could respond correctly by monitoring for either acoustic or phonemic identity. Participants' target detection responses were coded as hits, misses, false alarms, and correct rejections for signal-detection analyses.

There were 4 runs of the speech perception task, with each run lasting 15 min with short periods of rest between scanning runs. In each run, stimuli were presented in 35-s blocks grouped by acoustic form (clear, NV, SW) in which each syllable was presented once by each speaker, along with 2 additional events (chosen quasi-randomly) which constituted either a) 2 additional silent trials/TRs, b) 1 silent trial/TR and 1 repetition trial, or c) 2 repetition trials (see Fig. 1*C*). Following each block, there was 1 silent trial/TR (2.5 s) such that each block started 37.5 s (15 scans) after the start of the last block. There were 8 blocks of each acoustic form (clear, NV, SW) within a run, and hence a total of 24 blocks per run lasting a total of 15 min (see Fig. 1*E*). The order of the acoustic form blocks was randomized in triplets so that at maximum there could be no more than 2 consecutive blocks of the same acoustic form. The order in which the acoustic forms were played out was counterbalanced across subjects. Due to unexpected buffering delays in audio playback in E-Prime, stimulus timings occasionally drifted out of synchrony with scanner acquisitions, this occurred in 1 of the 4 runs for 4 of the participants. For these participants, the data associated with these runs were not analyzed.

### Univariate Analysis

Data were analyzed using SPM8 (http://www.fil.ion.ucl.ac.uk/spm, last accessed June 15, 2015). Preprocessing and statistical analyses were conducted separately for the speech production and perception tasks. For both tasks, the first 6 volumes of each run were removed to allow for T1 equilibrium effects. Scans were realigned to the first EPI image. The structural image was co-registered to the mean functional image, and the parameters from the segmentation of the structural image were used to normalize the functional images that were resampled to 2 × 2 × 2 mm. The realigned normalized images were then smoothed with a Gaussian kernel of 8-mm full-width half maximum to conform to the assumptions of random field theory and improve sensitivity for group analyses. Data were analyzed using general linear models as described later with a 128-s high-pass filter and AR1 correction for auto-correlation.

For the speech production model, the visual onset of each of the 6 syllables was modeled with a canonical hemodynamic response function as an event-related response separately for covert and overt naming trials, in addition to 6 movement regressors of no interest and the session mean. The rest condition provided an implicit baseline. Contrast images from the first-level model (the average of all syllable types for covert and overt reading trials, and the difference between covert and overt trials) were taken forward to second-level group analyses and entered into one-sample *t*-tests using the summary statistic approach.

For the speech perception model, auditory events were modeled with a canonical hemodynamic response function as an event-related response with each of the 36 unique stimulus types modeled separately. Additional columns were added to model hits, misses, and false alarms for the repetition detection task. Items entered into hit and miss events were excluded from the 36 stimulus conditions, but false alarms were specified as a stimulus condition event and an inadvertent button press. This ensured that the same number of events was specified for each of the 36 stimulus conditions and removed neural activity associated with the button press. In addition to these 39 conditions in each run, 6 movement parameters and the means of each run were added as regressors of no interest. Contrast images from the first-level model were entered into one-sample *t*-tests for group analyses. All statistical parametric maps and statistics reported in tables are thresholded at voxelwise level of *P*<0.001, with *q* < 0.05 false discovery rate (FDR) correction at the cluster level (Table 1).

**Table 1:** Description of activation in MNI coordinate system

Location	*X*	*Y*	*Z*	Extent	Z-value
Univariate effect of degradation [((NV + SW)/2) > CL]					
Left inferior frontal gyrus (p. Opercularis)	−54	10	20	555	3.92
Left precentral gyrus	−52	8	30		3.86
Left precentral gyrus	−46	2	28		3.80
Multivariate acoustic form coding					
Left posterior STG	−63	−33	15	392	4.94
Left mid-STG	−57	−12	4		4.62
Left mid-anterior STG	−54	−6	−4		4.37
Right posterior STG	54	−42	15	254	4.13
Right anterior STG	57	12	−8		3.99
Right supramarginal Gyrus	54	−36	26		3.83
Multivariate syllable identity coding					
Left mid-posterior left STG	−63	−24	8	303	4.59
Left mid-STG	−48	−15	0		4.38
Left posterior STS	−57	−39	8		4.21
Right posterior STG	69	−30	11	172	4.44
Right posterior STG	57	−27	8		4.19
Right mid-STG	63	−18	0		3.99
Left precentral gyrus	−51	0	41	83	4.39
Left postcentral gyrus	−60	−3	38		3.97

Note: multivariate coordinates are reported at the voxel size of acquisition (3 × 3 × 3.75 mm) and rounded to the nearest millimeter.

### Multivariate Analysis

Multivariate analyses were conducted on data following realignment, but without normalization or smoothing, generating statistical maps in each participants' native space. The structural image was co-registered to the mean of the EPI images, and the parameters from the segmentation of the T1 image were estimated in order to calculate the transformation from native to MNI space for each participant for use in group analyses. Each unique stimulus condition was modeled with a separate regressor for events occurring within odd and even blocks within each run (36 stimulus types in even-numbered blocks, and 36 stimulus types in odd numbered blocks, plus hits, misses, and false alarms as before). By comparing events in even and odd blocks, we can assess the similarity between responses to the same or different stimulus without differences in the delay between these replications, thereby reducing the influence of physiological noise on neural similarity measures. In all other respects, the design matrices were the same as used for the univariate analysis, including events for hits, misses, and false alarms, 6 movement parameters, and the mean for each scanning run. This generated 82 regressors per run and 328 regressors in total for the experiment. T-statistic maps were generated for the contrast of each stimulus condition (relative to un-modeled, silent periods) for odd and even blocks, creating 72 statistical maps. As T-maps combine the effect size weighted by error variance for a modeled response, they provide high-classification accuracy in multivariate analyses since results are not unduly influenced by large, but highly variable response estimates (Misaki et al. 2010).

These T-statistic images were then submitted to RSA (Kriegeskorte et al. 2008) using the RSA toolbox (Nili et al. 2014). In RSA, the observed similarity of neural patterns associated with different stimulus conditions is compared with a hypothetical model of how those conditions are related to one another. This allows us to test competing accounts of how information might be represented in the brain. At a practical level, it involves constructing a neural representational matrix in which each cell contains a similarity value (in this case, a correlation coefficient) reflecting the similarity between the neural pattern associated with one condition and every other. The degree of similarity between the neural representational matrix and a matrix expressing a hypothetical model is then calculated. RSA can be conducted within a region of interest (ROI) or using a searchlight approach (Kriegeskorte et al. 2006). In an ROI analysis, the neural representational matrix is calculated from a circumscribed anatomical or functional region. In a searchlight analysis, the neural patterns are extracted in turn from each voxel and its surrounding neighborhood, with this repeated across the whole brain. The similarity between the model and the neural data within each searchlight is then returned to the voxel at the center of the searchlight for further analysis.

In searchlight RSA, data were extracted from native space T-statistic maps by masking each map with a whole-brain mask generated during the model estimation step to restrict the analysis to voxels within the brain (mean number of voxels = 34 898). Spherical searchlights with a radius of 8 mm (65 voxels of 3 × 3 × 3.75, 2194 mm^3^) were extracted from the brain volume in searchlights with 2 or more voxels. At each searchlight location, the data from the 72 conditions were Pearson product-moment correlated with every other condition to generate a matrix of correlation coefficients reflecting the between-condition similarity of neural responses. These values were then Spearman-rank correlated with a set of models reflecting different predictions for the similarity structure of neural responses in different conditions. These models were expressed as a set of matrices, with values of +1 expressing increased similarity between particular conditions, and values of −1 expressing relative dissimilarity. Some elements of the model matrices were excluded from contributing to the correlation as they were not relevant to the hypothesis being tested (N/A values in Figs 3 and 5). The resulting correlation coefficient reflecting the correlation between predicted and observed similarity patterns was converted to a *z*-value using a Fisher transform so as to conform to statistical assumptions (normality) required for second-level parametric statistical tests on the resulting images. These Fisher transformed whole-brain maps were normalized to MNI space using the parameters estimated from segmentation. Each Fisher transformed map was then submitted to a second-level one-sample *t*-test to identify voxels in which the correlation between predicted and observed similarity values was >0. All group statistical maps from multivariate analyses were thresholded at an uncorrected peak level *P* < 0.001, with a *q* of <0.05 FDR correction at the cluster level.

Additional follow-up RSA analyses were conducted within anatomically or functionally defined ROIs. These ROIs were defined in MNI space and inverse-normalized into the native space of each participant using the parameters derived from segmentation. ROIs for bilateral PAC were determined using the cyto-architectonically defined regions TE1.0, 1.1, 1.2 (Morosan et al. 2001; available via the SPM Anatomy toolbox). Functionally defined ROIs were constructed either 1) using the independent motor localizer task or 2) using regions identified by leave-one-subject-out RSA searchlight maps to avoid statistical bias (Esterman et al. 2010).

## Results

### Behavioral Responses

A repeated-measures one-way ANOVA with 3 levels (CL, NV, and SW) was conducted on the d′ scores for one-back detection performance in the scanner. This showed that there was a significant difference in detection accuracy as a function of stimulus type (*F*_2,32_ = 22.484, *P* < 0.001, *η*^2^ = 0.584) (see Fig. 1*B*i). Follow-up repeated-measures *t*-tests using a SIDAK correction showed that d′ scores were higher for monitoring repetitions of CL (mean = 3.48) as compared with NV (mean = 2.95, *P* < 0.001) and SW speech (mean = 2.82, *P* < 0.001), but there was no significant difference in monitoring accuracy between the degraded conditions (*P* = 0.619).

A paired *t*-test conducted on the post-scanning syllable identification scores after conversion to rationalized arcsine units (Studebaker 1985) showed there to be a small but significant difference in the intelligibility of the 2 degradation types (*t*_16_ = 3.635, *P* = 0.002), with NV (mean proportion correct = 0.95) shown to be more intelligible than SW speech (mean = 0.88) (Fig. 1*B*ii).

### Univariate fMRI Analysis

#### Speech Perception Task

**Figure 2.**
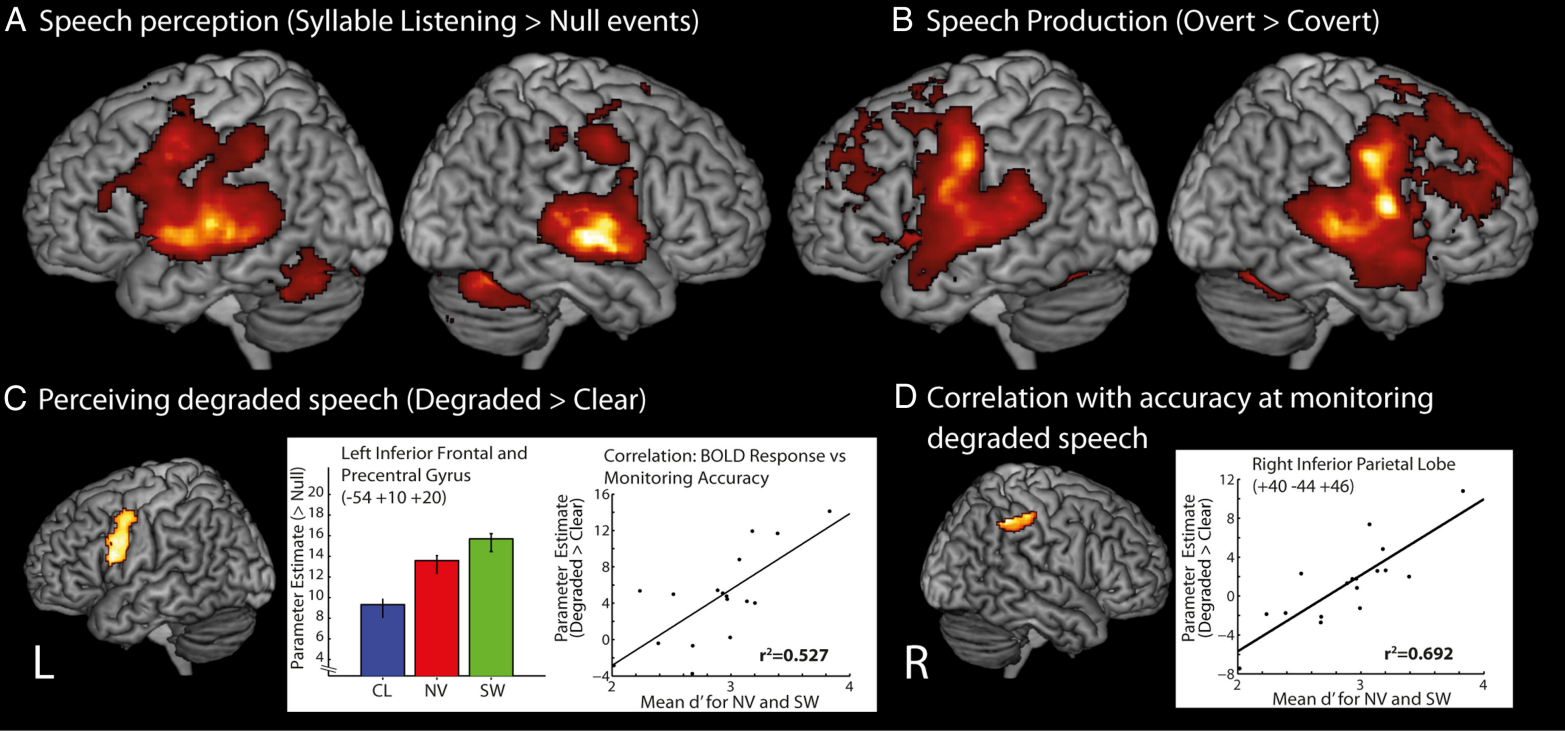
(*A*) Speech perception network: [Syllable listening > Null events] rendered onto a canonical brain image. (*B*) Speech production network: [Overt > Covert]. (*C*) Effect of degradation: [((NV + SW)/2) > CL]. Response plot shows parameter estimates from the peak voxel in the LIFG and premotor cluster with error bars suitable for repeated-measures comparisons (Loftus and Masson 1994), and a scatter plot of the correlation between the BOLD response from the peak voxel and the d′ scores for the identification of repetitions of degraded syllables. (*D*) Whole-brain correlation relating d′ scores for degraded syllables and brain activity for degraded speech, with a scatter plot of the data within the Inferior Parietal Lobe region showing a significant correlation (for purpose of illustration not for effect size inference). All results presented at *P* < 0.001 peak level uncorrected, *q* < 0.05 cluster-level FDR corrected.

Contrasting the response to hearing syllables with the response to silent rest periods revealed bilateral activation clusters in the STG, middle temporal gyrus (MTG), the cerebellum and the pre- and post-central gyrus, and left lateralized activation in the inferior temporal gyrus, inferior parietal and more ventral frontal cortex (including the inferior frontal gyrus) (Fig. 2*A*). A cluster responding more to degraded than to clear speech [((NV + SW)/2)) - CL] was found that extended across the left inferior frontal (pars Opercularis) and precentral gyrus (Fig. 2*C*). Analysis with a second-level covariate showed that activity in a cluster within the right inferior parietal lobule (peak centered at [40 −44 46], *r*^2^ = 0.692, *P* < 0.001, extent = 477 voxels) extending into the post-central gyrus correlated with individual differences in participant accuracy on the one-back detection task for the degraded conditions (the average d’ for NV and SW) at a whole-brain level (Fig. 2*D*). We also conducted a correlation analysis on beta values extracted from the peak of the inferior frontal and precentral gyrus cluster associated with degraded speech perception: activity within this cluster was correlated with individual differences in detection of repeated degraded syllables (*r*^2^ = 0.527, *P* = 0.001).

#### Speech Production Task

In order to identify brain regions selectively engaged by the somatosensory and motor processes involved in producing speech, we contrasted the activity associated with producing syllables out loud with producing the same syllables covertly (overt > covert speech production). This identified widespread activation within bilateral prefrontal regions extending to the pre- and post-central gyri (the left hemisphere peak was found at −50 −12 36, *Z* = 5.89), as well as parietal and temporal cortex presumably associated with monitoring speech output (cf. Guenther et al. 2006 and Hickok et al. 2011, the frontal operculum, insulae, and cerebellum (Fig. 2*B*). Activation in these somatomotor regions overlapped with many of the regions that responded during perception (the conjunction null: [speech perception > rest] ∩ [overt > covert production]; Nichols et al. 2005); direct overlap was found in temporal and frontal cortex (including the pre and post central gyrus) and the cerebellum. Overlap is also observed between brain regions activated during speech production and the precentral gyrus region activated during perception of degraded compared with clear syllables (as depicted in Fig. 4*A*i, this consists of 90 2-mm isotropic voxels with a center of mass at [-46 0 32]).

### Multivariate fMRI Analysis

#### Syllable Identity and Acoustic Form Representations

**Figure 3.**
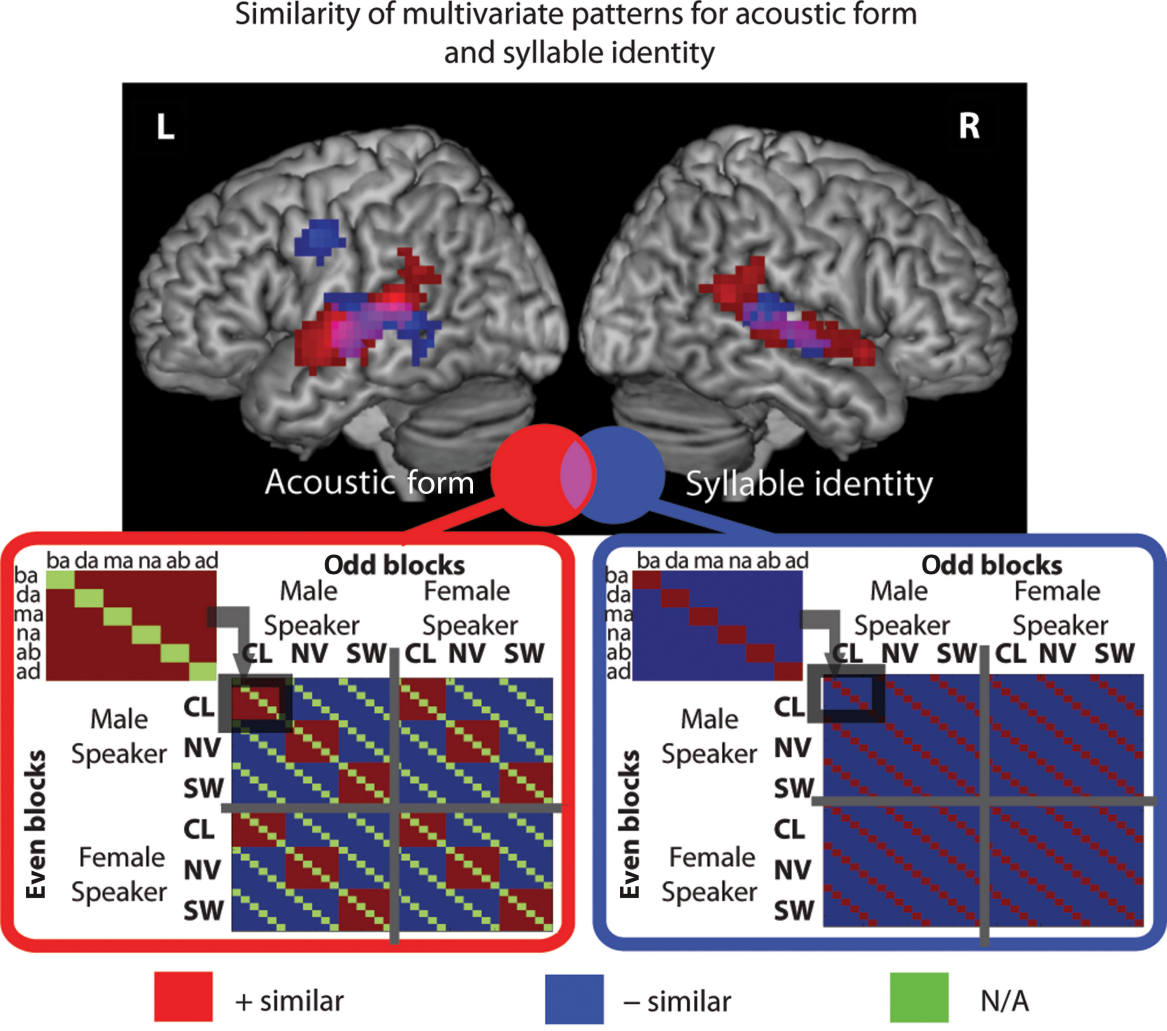
Similarity of multivariate patterns for the acoustic form (left) and syllable identity (right) representational similarity analysis models. RSA models express expected differences in voxel pattern similarity between neural responses to different syllables presented in different acoustic forms in odd and even blocks of trials. Red tiles indicating increased similarity between pairs of neural patterns, blue tiles indicating decreased similarity, green tiles indicating comparisons that were removed from analysis as not applicable (N/A). Results of searchlight RSA for these 2 models are shown rendered onto a canonical brain image. All results presented at *P* < 0.001 peak level uncorrected, *q* < 0.05 cluster-level FDR corrected.

Two RSA models were initially examined. The first model (Acoustic Form) tested for responses in which similarity was driven by the surface acoustic form of the syllables (Fig. 3, left). Hence, this model tests for regions in which similarity was increased for syllables of the same acoustic form (CL to CL, or NV-NV, SW-SW, red elements indicating greater predicted similarity) and decreased for syllables of a different form (CL to NV, CL-SW, SW-NV, blue elements indicating reduced predicted similarity). In this model, we removed the possibility that similarity could be driven by responses to the underlying identity of the syllable by excluding identical underlying syllables from each of the comparisons (green elements). Using a whole-brain searchlight analysis, we identified several clusters of spotlight locations in which pattern similarity was sensitive to acoustic form independent of syllable identity (Fig. 3, red rendering). Clusters were located in the left and right STG, extending into PAC and the supramarginal gyrus. Within the STG clusters, in the left hemisphere, 6.5% of the cluster was found in TE 1.2, and in the right hemisphere, 7.9% and 4.1% were found in TE 1.0 and 1.2, respectively.

The second model (Syllable Identity) examined responses in which similarity was driven by the identity of the syllables (Fig. 3, right). This model tested for regions in which the response to syllables with the same underlying phonological identity (e.g., a /ba/ most similar to /ba/) was more similar (red elements) than for syllables with a different phonological identity (e.g., /ba/ to /da/, /ma/, /na/, /ab/ or /ad/) (blue tiles), irrespective of the surface acoustic form of those syllables (i.e., we predict increased similarity for /ba/ to /ba/ even if these 2 syllables were produced by a different speaker and presented in a different acoustic form). Using a whole-brain searchlight analysis, we found clusters of spotlight locations in which pattern similarity was greater for syllables of the same identity than for syllables with different identities (Fig. 3, blue rendering). These clusters were found within bilateral mid-posterior STG and MTG extending into the STS and the PAC. In the left hemisphere, 4.9% and 2.7% of the cluster extended into TE1.0 and 1.2, and in the right hemisphere, 0.7% of the cluster extended into TE1.1. Most striking, though, was a significant cluster in the pre- and post-central gyrus that showed significantly greater similarity for syllables of the same identity.

**Figure 4.**
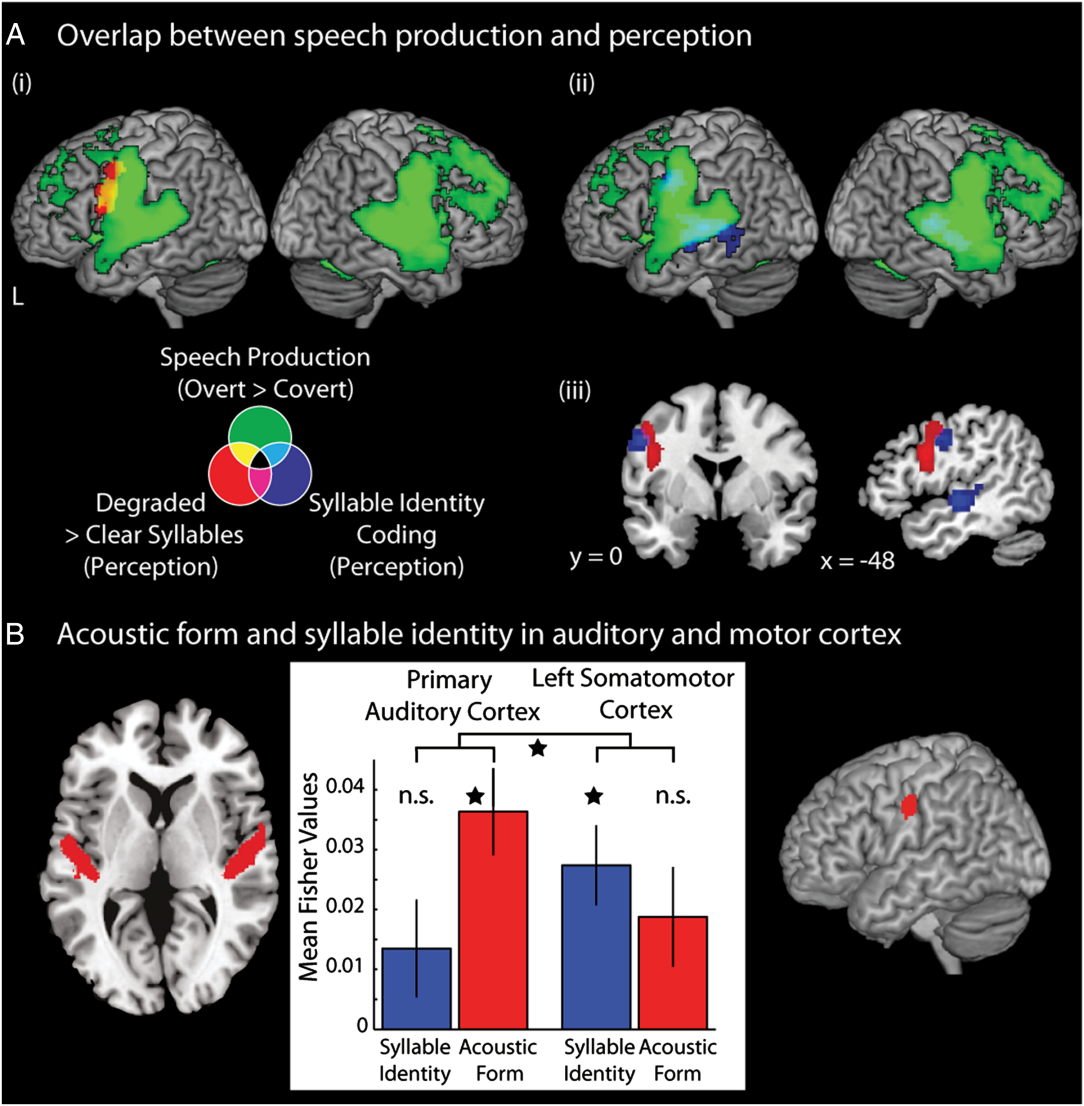
(*A*) Univariate activity associated with speech production: [Overt > Covert] (green), signal degradation during speech perception: [((NV + SW)/2) > CL] (Red), and multivariate syllable identity coding (blue) displayed on a canonical brain image. Note that multivariate decoding maps were resampled to 2-mm isotropic voxels to illustrate and quantify voxel-to-voxel overlap between univariate and multivariate results. i) Overlap between neural responses to signal degradation and speech production, ii) overlap between neural coding of syllable identity and neural responses during speech production and iii) coronal and sagittal sections showing the adjacent location of activation of responses to speech degradation during perception and syllable identity coding. (*B*) Comparison of the similarity response in the bilateral PAC and the left somatomotor cortex for the syllable identity and acoustic form models. Bar graphs show similarity response in these regions with error bars showing within-subject standard error (Loftus and Masson 1994). All results presented at *P* < 0.001 peak level uncorrected, *q* < 0.05 cluster-level FDR corrected.

Overlapping the statistical maps for syllable identity coding with regions activated by degraded speech and by speech production in the univariate analyses showed neural convergence within left somatomotor cortex (Fig. 4). This reflected a direct voxel-by-voxel overlap between regions associated with syllable identity coding and speech production (Fig. 4*A*ii). This consisted of 73 voxels (2-mm isotropic since multivariate maps were resampled in order to quantify voxel overlap with univariate maps at the same resolution) in the left precentral gyrus (center of mass: [−54 −4 36], rounded to the nearest integer coordinate), 355 voxels in the left temporal cortex (center of mass: [−56 −22 2]), and 141 voxels in the right temporal cortex (center of mass: [58 −24 4]). Unlike the overlap between speech production and degraded speech perception (reported previously, and depicted in Fig. 4*A*i), there was no direct voxel-by-voxel overlap within somatomotor cortex between spotlight locations showing significant syllable identity coding and brain regions activated by the perception of degraded compared with clear speech. However, these 2 significant clusters were located in very close proximity to each other (Fig. 4*A*iii)—with degraded speech perception activating an inferior frontal and precentral gyrus region slightly more rostral to the somatomotor regions that show syllable identity coding.

We then conducted similarity analyses using the same acoustic form and syllable identity models within ROIs in anatomically defined bilateral PAC and within the region of left somatomotor cortex activated by speech production in the localizer task (ROI shown in Fig. 4*B*). Comparison of neural similarity measures in these ROIs allows us to compare representations of heard speech in brain regions associated with speech production and in primary auditory regions. The speech production ROI was defined by thresholding the statistical map for [Overt > Covert] speech production with a stringent *P* < 0.005 voxel wise FWE correction to restrict the cluster to primary somatosensory and motor cortex. The peak of this cluster was at [−50 −12 36] and consisted of 144 voxels (at 2 × 2 × 2 mm voxel size) spanning both the pre- and post-central gyrus. This region significantly overlapped with the cluster in the precentral gyrus that coded syllable identity in our study and with somatomotor regions previously implicated in speech perception (Wilson et al. 2004; Pulvermüller et al. 2006).

One sample *t*-tests showed that the response of PAC showed greater pattern similarity for pairs of syllables presented in the same acoustic form irrespective of syllable identity (significant fit to the acoustic model [*t*_16_ = 3.557, *P* < 0.003]). However, pattern similarity was no greater for pairs of syllables with the same underlying syllable identity (i.e., no significant fit to the syllable identity model, *t*_16_ = 1.506, *P* = 0.152). Conversely, the response in somatomotor cortex showed greater pattern similarity for the same underlying syllable (as predicted by the syllable identity model, *t*_16_ = 3.296, *P* = 0.005), but not for syllables presented in the same acoustic form (*t*_16_ = 1.748, *P* = 0.100). A 2 × 2 repeated-measures ANOVA with factors: region (PAC, somatomotor) and model (acoustic, identity) demonstrated no main effects of region (*F*_1,16_ = 0.146, *P* = 0.708, *η*2 = 0.009) or model (*F*_1,16_ = 0.304, *P* = 0.589, *η*2 = 0.019), but a significant cross-over interaction (*F*_1,16_ = 6.016, *P* = 0.026, *η*2 = 0.273) reflecting a double dissociation of responses to acoustic and syllable identity information in PAC and somatomotor cortex (Fig. 4*B*). These results show PAC and somatomotor cortex sit at opposite ends of the speech processing hierarchy: during a simple 1-back detection task, brain regions involved in speech production show activation patterns consistent with representing the underlying identity of heard syllables but not their acoustic form. Conversely PAC shows activation patterns consistent with a representation of the surface acoustic form of speech, but not underlying syllable identity.

#### Graded Abstraction of Syllable Identity Representations

**Figure 5.**
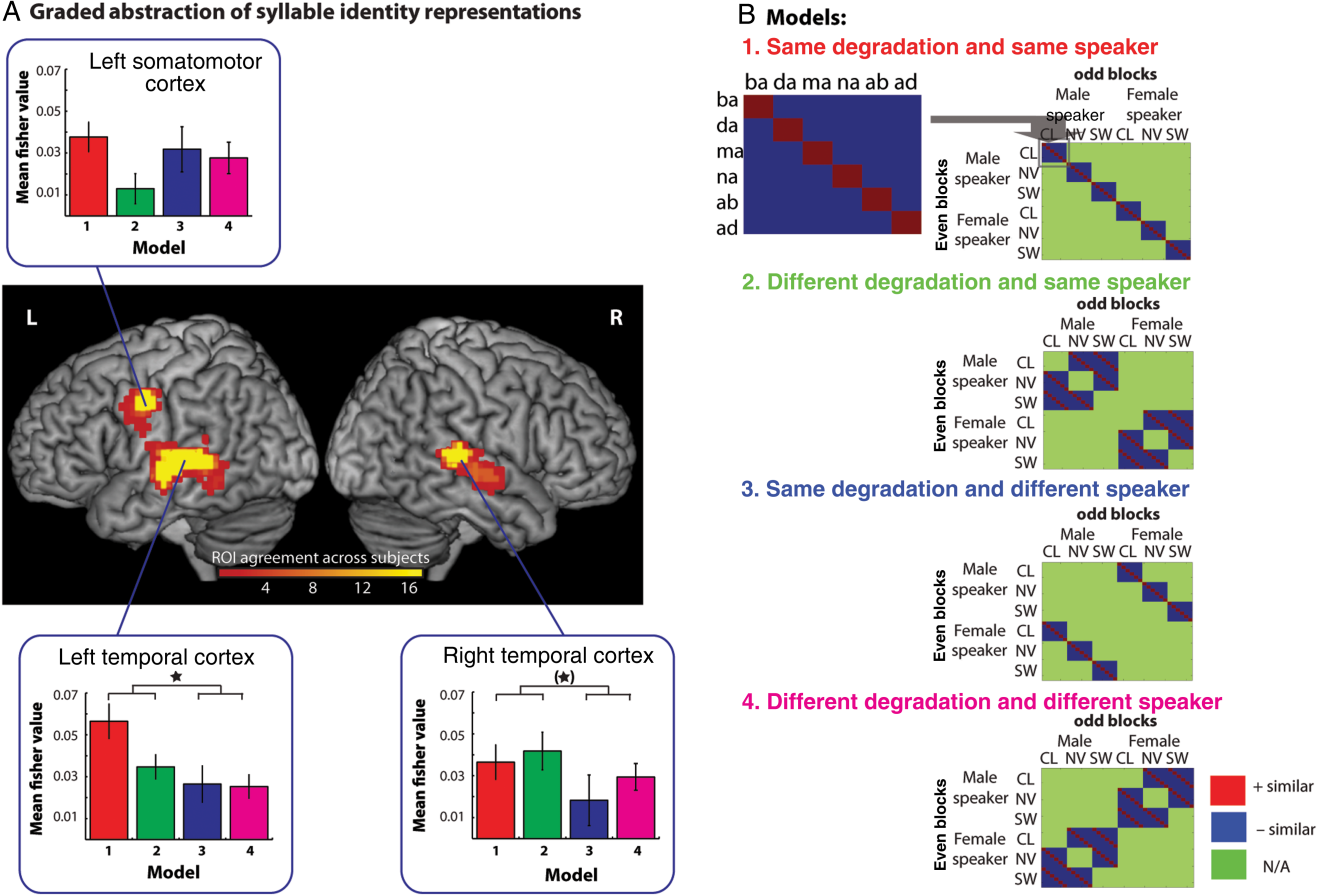
Hierarchical abstraction of syllable encoding in the left precentral gyrus and the left and right temporal cortex. (*A*) Brain rendering showing the overlap between the leave-one-participant-out ROIs used in the analysis. These regions showed a significant fit to the full syllable identity coding model (Fig. 3, right panel) but are defined for each participant using data from all other participants. This ensured that follow-up tests comparing different submodels are statistically independent of the data used to define the ROI. We differentiated the response in these regions by testing for 4 component models shown in (*B*). These test for similarity driven for identical syllables presented in the Same Degradation-Same Speaker (Model 1), Different Degradation-Same Speaker (Model 2), Same Degradation-Different Speaker (Model 3), and Different Degradation-Different Speaker (Model 4, the most abstract form of similarity tested in the current design). Red tiles indicate cells in which increased similarity is expected, blue tiles indicate decreased similarity, and green tiles are comparisons removed from analysis. Bar graphs show similarity responses for each of these 4 models in each ROI with within-subject error bars representing one standard error of the mean suitable for repeated-measures comparisons (Loftus and Masson 1994).

Neural representations of syllables detected by the identity model could reflect several different levels of abstraction from the surface acoustic form of the speech signal. We can characterize the degree of abstraction of neural representations in temporal and somatomotor regions by differentiating identity coding in 4 submodels that comprise a 2-by-2 factorial crossing of whether syllables come from the same or different speaker and are presented in the same or different acoustic form (i.e., degradation). The 4 models can be described thus:

Model 1. Same Degradation and Same Speaker (Fig. 5, Red) quantifies increased similarity for the same acoustic token, that is, syllables presented in the same acoustic form (CL, NV, or SW) and spoken by the same speaker (male or female). This model therefore expresses the lowest degree of abstraction. Even an unmodified acoustic representation would be expected to show similarity of this type. Note, however, that since comparisons are made between odd- and even-numbered blocks in each scanning run, this model still requires greater than chance similarity between neural responses to different presentations of the same acoustic token.
Model 2. Different Degradation and Same Speaker (Fig. 5, green) quantifies increased similarity for the same syllable presented in a different acoustic form (CL to NV, CL to SW, etc.) but spoken by the same speaker. This would identify regions in which, for example, a NV /ba/ from the male speaker was more similar to a SW /ba/ than to a SW /da/ from the male speaker.
Model 3. Same Degradation and Different Speaker (Fig. 5, Blue) quantifies increased similarity for the same syllable presented in the same acoustic form but spoken by a different speaker. This model would highlight regions in which, for example, a NV /ba/ from the male speaker was more similar to a NV /ba/ from the female speaker, than to a NV /da/ from the female speaker.
Model 4. Different Degradation and Different Speaker (Fig. 5, Pink) quantifies increased similarity for the same syllable presented in a different acoustic form and spoken by a different speaker. This model would identify regions in which, for example, a NV /ba/ from the male speaker was more similar to a SW /ba/ from the female speaker than a SW /da/ from the female speaker. This model therefore expresses the greatest degree of abstraction since syllable identity is represented in a manner that is invariant to changes to the speaker and acoustic form of each syllable.

We ran follow-up ROI similarity analyses with each of the 4 models described earlier, within the 3 regions identified by the full identity model in the left and right temporal cortex and the left precentral gyrus (Fig. 3). In order to avoid statistical bias (given that each of these 4 models is a subset of the full identity model), ROIs were defined using a leave-one-subject-out method (Esterman et al. 2010). That is, to identify an ROI for Participant 1, we re-estimated the random effects *t*-test using the whole-brain searchlight maps for the full identity model with Participants 2 to 17. We thresholded these maps at a *P* < 0.004 (uncorrected) to extract clusters in the left and right temporal cortex and the left somatomotor cortex (in the pre- and post-central gyri); this threshold was chosen as it ensured that we could identify 3 discrete clusters in all leave-one-out permutations and that these clusters did not extend to other anatomical regions. This generated 17 subtly different ROIs, which provided statistically independent regions for testing differences between the 4 models described earlier. The heat map and color bar in Figure 5 show the mean location of these ROIs across subjects.

The most abstract representation of syllable identity is indicated by Model 4 (greater similarity for identical syllables despite being spoken by different speakers and presented in a different acoustic form). This model was significant in all 3 regions (all *P* ≤ 0.024). A one-way ANOVA examining differences between the regions in their response to this model was not significant (*F*_2,32_ = 0.026, *P* = 0.974, *η*2 = 0.002). Hence, all 3 regions achieve a substantial degree of invariance to speaker and degradation changes in representing the identity of spoken syllables. However, this finding need not imply that there is no influence of speaker or degradation changes on neural representations of syllable identity. To assess this, we conducted a 3 × 2 × 2 repeated-measures ANOVA with the factors region (left somatomotor, left temporal, right temporal), degradation type (same/different degradation type), and speaker (same/different speaker) on the Fisher transformed values. This showed a main effect of speaker (*F*_1,16_ = 6.871, *P* = 0.019, *η*2 = 0.300), indicating greater similarity for syllables spoken by the same than for different speakers. No other main effects or interactions reached statistical significance. We then conducted 2 × 2 ANOVAs with the factors degradation (same/different) and speaker (same/different) within each of these 3 regions. This showed that the effect of speaker on response pattern similarity was reliable in the left temporal cortex (*F*_1,16_ = 6.306, *P* = 0.023, *η*2 = 0.283) and identified a similar trend in the right temporal cortex (*F*_1,16_ = 3.308, *P* = 0.088, *η*2 = 0.171), but there was no effect of speaker changes on similarity in somatomotor cortex (*F*_1,16_ = 0.340, *P* = 0.568, *η*2 = 0.021). A 2 × 2 × 2 ANOVA including just the left somatomotor and left temporal lobe clusters showed a marginal region × speaker interaction (*F*_1,16_ = 3.618, *P* = 0.075, *η*2 = 0.184). While the absence of any significant interaction with region in the overall ANOVA limits the strength of conclusions that can be drawn concerning differences between these regions in their representations of spoken syllables, these findings are suggestive of greater sensitivity to speaker identity in temporal than in frontal regions.

To further test the response in somatomotor cortex, we used an ROI defined on the basis of activity in the independent speech production run (ROI shown in Fig. 4*B*). In this ROI, we tested the most abstract identity coding model (Model 4—different speaker and different degradation type) which was shown to be significant (*t*_16_ = 3.820, *P* = 0.002). We further conducted a 2 × 2 ANOVA with degradation type (same/different) and speaker (same/different) as factors within this region. This showed there to be no significant main effects or interactions (all *P* > 0.1) suggesting coding of abstract syllable identity irrespective of degradation or speaker identity.

A further question with regard to somatomotor responses concerns whether representations of syllable identity are specific to degraded speech. This might be anticipated based on the increased univariate activity observed for degraded compared with clear speech (Fig. 2*C*). We therefore computed 2 further models, in which we assessed syllable identity representations for clear speech (the combination of CL to CL, CL to NV, and CL to SW) and for degraded speech (NV to NV, SW to SW, and NV to SW). For the left precentral gyrus ROI (defined by a leave-one-out procedure as mentioned earlier), both of these models showed significant representation of syllable identity (clear speech [*t*_16_ = 2.587, *P* = 0.020]; degraded speech [*t*_16_ = 3.723, *P* = 0.002]), and there was no evidence of a difference between these 2 models (*t*_16_ = 1.131, *P* = 0.275). For the left somatomotor cortex ROI (defined from the speech production run, Fig. 4*B*), only the clear speech model showed significant representation of syllable identity (clear speech [*t*_16_ = 3.826, *P* = 0.001]; degraded speech [*t*_16_ = 1.614, *P* = 0.126]), but there was no evidence of a difference between these 2 models (*t*_16_ = 1.450, *P* = 0.166). These results indicate reliable coding of syllable identity in somatomotor regions even for clear speech.

### Structure of Syllable Identity Representations

**Figure 6.**
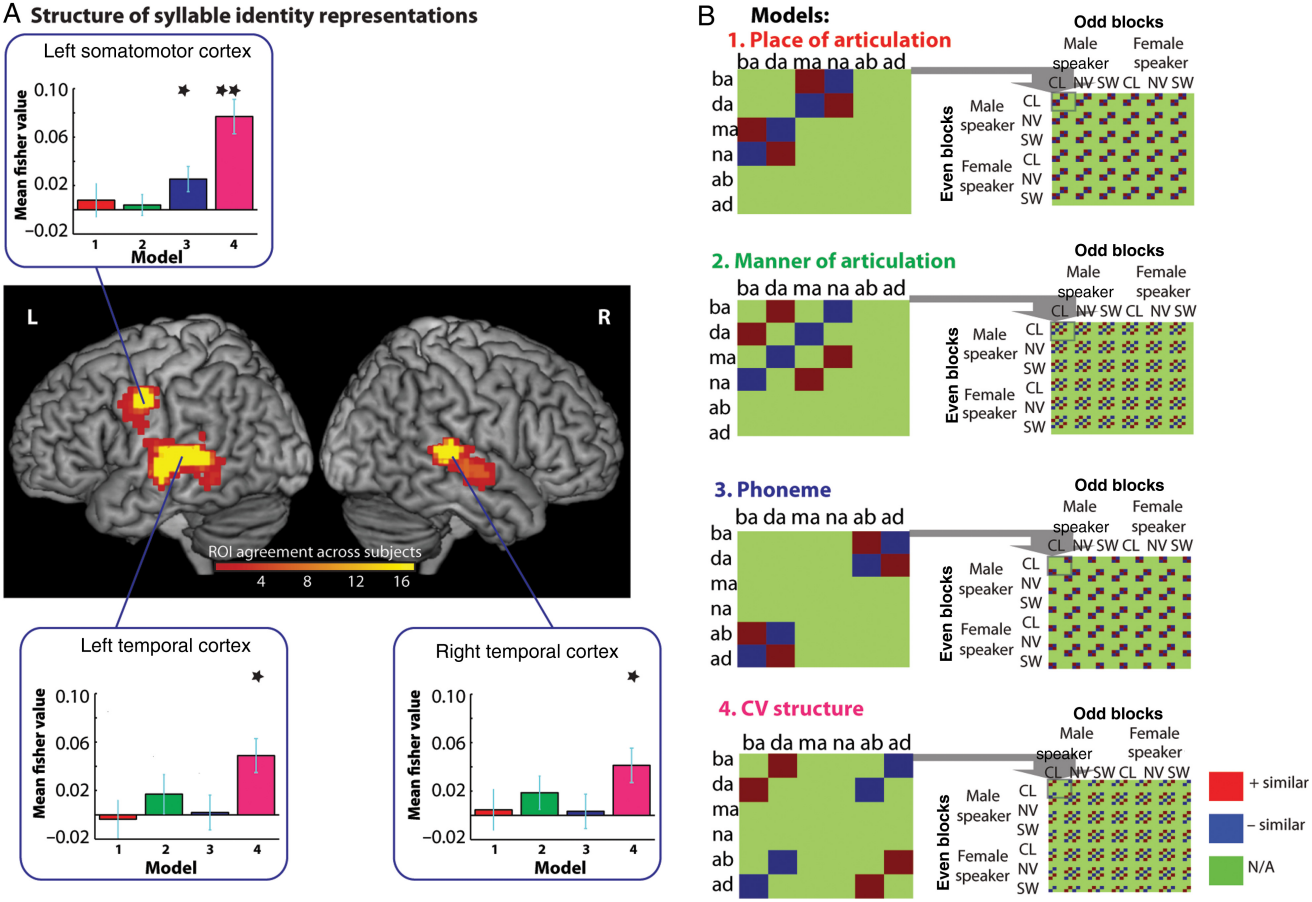
Structure of syllable identity representations within the left precentral gyrus and the left and right temporal cortex. (*A*) Brain rendering showing the overlap between the leave-one-participant-out ROIs used in the analysis. We further differentiated the response in regions shown to encode syllable identity to identify the additional structure of syllable identity representations (*B*) within these regions. These test for similarity driven by the place of articulation feature (Model 1) and manner of articulation feature for consonants (Model 2), phoneme identity (Model 3) and CV vs. VC syllable structure (Model 4). Red tiles indicate cells in which increased similarity is expected, blue tiles indicate decreased similarity, and green tiles are comparisons removed from analysis. Bar graphs show similarity responses for each of these 4 models in each ROI as plotted in Figure 5.

Having demonstrated representations of syllable identity, we now assess the nature of syllable identity representations at different levels of the processing hierarchy. We do this by considering similarity between pairs of non-identical syllables that nonetheless shared articulatory features, phonemes, or Consonant-Vowel syllable structure. To do this, we compared the similarity of non-identical syllable pairs that shared a specific feature (e.g., bilabial place of articulation is common to both the oral stop /ba/ and the nasal stop /ma/) and pairs that differed in that feature (e.g., /ba/ and /na/). These models were specified for all combinations of same/different speaker and same/different degradation and tested within the same leave-one-subject-out ROIs used before; these ROIs and models are shown in Figure 6 and described below:

Model 1. Place of articulation feature model (Fig. 6, Red) quantifies increased similarity for pairs of CV syllables that share the same place of articulation compared with pairs that differ in place of articulation. For example, this model specifies that the bilabial consonant /ba/ is more similar to bilabial /ma/ than to the alveolar /na/, whereas alveolar /da/ is more similar to alveolar /na/ than bilabial /ma/. Note that these comparisons involve changes in manner of articulation; /ma/ and /na/ are both nasal consonants whereas /ba/ and /da/ are oral consonants.
Model 2. Manner of articulation feature model (Fig. 6, Green) quantifies increased similarity for pairs of CV syllables that share the same manner of articulation compared with pairs that differ in manner. For example, this model specifies that the oral consonant /ba/ is more similar to the oral /da/ than to /na/ which is a nasal consonant, whereas nasal /ma/ is more similar to nasal /na/ than oral /da/. Note that both these comparisons involve changes in place of articulation: /ma/ and /ba/ are both produced with bilabial closure whereas /da/ and /na/ are both produced with alveolar closure.
Model 3. Phoneme model (Fig. 6, Blue) quantifies increased similarity between CV and VC syllables that share the same phonemes compared with pairs of syllables that contain different phonemes. For example, this model specifies that /ba/ is more similar to /ab/ syllable which shares 2 phonemes, than to /ad/ which only shares one phoneme. Conversely, /da/ is more similar to /ad/ than /ab/. Note that both of these comparisons involve changes to CV structure: /ab/ and /ad/ are VC structure syllables, whereas /ba/ and /da/ are CV syllables.
Model 4. CV structure model (Fig. 6, Pink) quantifies increased similarity for syllables that share the same CV structure compared with syllable pairs that have different structures. For example, this model specifies that a CV /ba/ is more similar to a CV /da/ than to a VC /ad/ whereas /ab/ is more similar to /ad/ than to /da/. Note that both of these comparisons involve changes to phoneme content, /da/ and /ad/ contain the same phonemes, as do /ba/ and /ab/.

The CV structure model was significant in all 3 regions (left precentral gyrus, *P* < 0.001, left temporal, *P* = 0.001, right temporal, *P* = 0.003). In contrast, the phoneme model was only significant in the precentral gyrus (*P* = 0.011) and neither of the feature models (place or manner of articulation) were significant in any region (all *P*s > 0.126). To assess between-region effects, we conducted a 3 × 4 repeated-measures ANOVA on the Fisher transformed values, with region (left precentral, left temporal and right temporal) and model (place, manner, phoneme, CV structure) as factors. This showed there to be a significant effect of model (*F*_3.48_ = 6.579, *P* = 0.001. *η*2 = 0.291), which was driven by stronger encoding of the CV structure model compared with all other models (all *P*s < 0.007), and a marginal region by model interaction (*F*_6,96_ = 1.973, *P* = 0.077, *η*_2_ = 0.110), with no main effect of region (*F*_2,32_ = 1.906, *P* = 0.165, *η*2 = 0.106). A further 2 × 4 repeated-measures ANOVA comparing responses in left temporal and precentral gyrus ROIs (with region and model as factors) showed there to be a marginal region by model interaction (*F*_3,48_ = 2.294, *P* = 0.090, *η*2 = 0.125) whereas the equivalent interaction was absent in comparison of the left and right temporal lobe (region by model interaction, *P* = 0.789). While these marginally significant interactions with region only support very tentative conclusions concerning differences between regions, follow-up comparisons suggest that the left precentral gyrus represents heard speech using phonemic or syllabic representations to a greater degree than the left temporal lobe region; simple comparison of the phoneme model shows a marginally significant reduction (*t*_16_ = 1.926, *P* = 0.072), as does the equivalent comparison for the CV structure model (*t*_16_ = 2.068, *P* = 0.055), whereas neither of the feature models show any change between region (both *P* > 0.377).

## Discussion

We used searchlight RSA to examine neural coding of spoken syllables that differed greatly in their acoustic form as a consequence of changing speaker and degrading the surface characteristics of speech. We have shown that multivariate patterns in bilateral superior temporal and left somatomotor cortex code the identity of syllables irrespective of substantial changes in auditory form. By comparing the degree to which multivoxel patterns in auditory, temporal, and somatomotor regions also code for surface acoustic characteristics, we provide evidence that these regions sit at different levels of a processing hierarchy that maps the variable acoustic forms of speech signals to more abstract representations of syllable identity. Indeed, we find evidence for a graded hierarchy of abstraction across the brain, such that anatomically defined PAC represents the surface acoustic form of speech (such as whether syllables are clearly spoken, NV or SW synthesized) irrespective of the identity of the syllables. At an intermediate level, bilateral temporal cortex encodes both syllable identity (including speaker information) and the surface acoustic form of the speech. The most abstract representations of syllables are found in somatomotor cortex, which represents syllable identity without encoding speaker differences and does not significantly differentiate the surface acoustic form of speech signals. In this region, there was some evidence that syllable identity representations were organized in terms of phonemic units and CV structure, and this was more apparent in somatomotor than temporal lobe regions. In a later section, we consider the role of abstract somatomotor representations in neural accounts of speech perception, a domain in which motor contributions are much debated. However, we first consider the implications of our findings for the hierarchical organization of speech perception.

### Hierarchical Organization of Speech Perception

Our study identified a hierarchically organized temporo-frontal network that contributes to speech perception. While hierarchical organization of auditory and speech networks has been suggested previously (Wessinger et al. 2001; Davis and Johnsrude 2003; Okada et al. 2010; Peelle et al. 2010; Evans, Kyong et al. 2014), our RSA analyses extend these findings by determining the degree of abstraction with which speech is represented within these networks. The lowest level of abstraction in neural representation was shown in anatomically defined PAC, which encoded surface acoustic forms (whether syllables were presented as SW, NV, or clear speech), but not the identity of syllables. This is consistent with univariate studies showing that neural responses associated with categorical perception and speech intelligibility are only found in regions beyond PAC (Binder et al. 2000; Scott et al. 2000; Zevin and McCandliss 2004; Dehaene et al. 2005; Liebenthal et al. 2005; Joanisse et al. 2007). Our results add to previous MVPA fMRI studies in which voxels within and close to Heschl's Gyrus can classify isolated vowels and CV syllables (Formisano et al. 2008; Obleser et al. 2010; Kilian-Hutten et al. 2011). Here, using a larger set of syllables (6 rather than 2 or 3) and more extreme acoustic changes, including artificial degradation, we observed that the response within anatomically defined PAC (alone) did not encode abstract syllable identity. Our results suggest that voxels in hierarchically higher regions of auditory belt and parabelt are more critical for representing the identity of speech sounds.

Searchlight analyses showed that multivariate patterns in bilateral temporal cortex encoded both the surface acoustic form and the abstract identity of syllables. Follow-up ROI analyses showed that these representations were also sensitive to between-speaker differences. This is consistent with univariate fMRI observations that peri-auditory regions of the STG respond to both intelligibility and surface acoustic characteristics of speech (Davis and Johnsrude 2003; Obleser et al. 2004, 2006b, 2008; McGettigan, Evans et al. 2012). Studies of vocal perception also suggest a contribution of peri-auditory STG regions (particularly in the right hemisphere) to processing speaker identity (Formisano et al. 2008; Latinus et al. 2013; Bonte et al. 2014). Many of the acoustic features that discriminate between speakers are removed by noise-vocoding and SW synthesis (for example, fundamental frequency and harmonic to noise ratio) but others remain (e.g., formant frequency dispersion [Latinus et al. 2013]). Our observation that speaker information is encoded alongside syllable identity information suggests that indexical as well as abstract representations of syllables are simultaneously encoded in lateral temporal regions. These combined representations might increase the redundancy of the encoded speech signal allowing more robust perception particularly in the difficult listening conditions studied here.

Whole-brain searchlight analyses also showed that representations of syllable identity were observed in a region that overlapped with both primary motor and somatosensory cortex, suggestive of integrated motor and somatosensory (somatomotor) representations of speech sounds (Guenther et al. 2006; Davis and Johnsrude 2007). These representations are markedly different from earlier perceptual stages, as confirmed by ROI analyses, which showed a significant cross-over interaction of acoustic and identity similarity in comparison with anatomically defined primary auditory regions. These observations provide novel evidence for abstract, non-acoustic coding of the identity of heard speech in somatomotor regions.

Our study also provides evidence that somatomotor regions support articulatory coding of syllables during speech perception. This evidence comes from three additional observations derived from univariate statistical analyses: 1) These somatomotor regions were also activated during overt compared with covert speech production, 2) adjacent motor and premotor regions were more active for degraded as compared with clearly spoken syllables, and 3) these regions show activity correlated with individual differences in monitoring of degraded speech. These findings show neural convergence such that premotor and somatomotor regions traditionally associated with speech production are also engaged during perception. These results extend previous fMRI studies that have shown increased activation within primary motor cortex to degraded compared with clear speech (Adank and Devlin 2010; Osnes et al. 2011; Hervais-Adelman et al. 2012) and motor or premotor coding of segment identity during phoneme identification tasks (Lee et al. 2012; Du et al. 2014). A parsimonious explanation of all these observations is that the highest-level, most abstract representations of heard syllables involve articulatory coding in somatomotor regions.

However, we also used RSA models to directly test for articulatory feature representations (representations of place and manner of articulation) as well as representations of phonemic units and syllable structure (CV vs. VC). To our surprise, we failed to observe evidence that representations of syllable identity were structured in terms of place or manner of articulation features either in somatomotor or lateral temporal regions. This is in contrast to a recent MVPA study by Arsenault and Buchsbaum (2015), which demonstrated encoding of place and manner within the auditory cortices (but not frontal regions), and a study reported by Correia (2015) in which featural representations are shown in both peri-auditory and motor regions. It remains unclear why we failed to demonstrate similar place and manner coding in our study; one suggestion may be that the degraded speech tokens used in our study permitted less rich articulatory/acoustic feature representations than the clear speech tokens used in these other studies. Alternatively, it might be that our use of a one-back detection task of speech focused attention on larger, syllabic rather than segmental units.

Our results did, however, show somatomotor encoding of syllables in terms of their CV structure and constituent phonemes. Furthermore, marginally significant between-region differences suggest that these representation were perhaps more reliable in somatomotor as compared with lateral temporal cortex. Our observation of phonemic representation within somatomotor cortex is consistent with the premotor activity that has been shown during speech segmentation and categorization tasks (Burton et al. 2000; Sato et al. 2009) that may be supported by dorsal stream connectivity between temporal and premotor cortex (Chevillet et al. 2013). We note, however, that our study only used a limited set of phonemes, and syllable structures. We therefore had only a limited opportunity to compare alternative models of speech representation in temporal and somatomotor cortex.

### The Role of Somatomotor Representations in Speech Perception

Our findings of somatomotor representations of syllable identity can inform neural accounts of speech perception in which motor contributions are much debated (Lotto et al. 2009; Scott et al. 2009; Pulvermüller and Fadiga 2010). At the same time, we acknowledge that there are limitations to the strength of inference that can be drawn from our results. No functional imaging study of healthy individuals can show that an activated brain region is necessarily required for speech perception; such evidence can only come from studies in which neural processing is perturbed due to a prior lesion or brain stimulation (Uddin et al. 2006; Devlin and Watkins 2007; Weber and Thompson-schill 2010). Existing evidence from brain stimulation suggests that somatopically specific neural stimulation of motor regions can impair speech perception (D'Ausilio et al. 2009; Möttönen and Watkins 2009), and yet, for a variety of reasons, these results remain controversial (Hickok et al. 2011). Our functional imaging data combined with multivoxel pattern analysis methods reveal that somatomotor representations of abstract syllable identities are activated during speech perception. Yet, this does not demonstrate their causal role in perception. However, we follow Weber & Thompson-Schill (2010) in arguing that activation of these representations in our participants is caused by them hearing speech. By considering what aspects of the listening situation tested here cause activation of somatomotor representations, we can provide additional constraints on the functional (but not necessarily causal) role of somatomotor regions in speech perception.

It has been argued previously that somatomotor responses to speech may reflect the acoustic properties of sounds and are not specific to speech (Scott et al. 2009). However, we have shown that multivariate patterns in somatomotor cortex represent the underlying identity of syllables despite marked differences in their surface acoustic form. It seems unlikely that our results are due to acoustic properties of speech, independent of our listeners' ability to hear these sounds as specific syllables. In our study, this depended on prior training with NV and SW syllables (Remez et al. 1981; Davis et al. 2005). We predict that we would not observe somatomotor coding of syllable identity for listeners who were not trained to perceive these syllables as speech or who could not accurately distinguish degraded syllables. However, this remains to be tested. Conversely, it seems unlikely that our results can be explained entirely by short-term learning effects for degraded speech. Participants received the majority of training with these sounds up to 7 days (and at least 48 h) prior to scanning and rapid within scanner perceptual learning for degraded speech is associated with inferior frontal rather than somatomotor activity (Eisner et al. 2010). Furthermore, we see equivalent coding of syllable identity for clear speech as for degraded speech in somatomotor regions. Hence, we propose that the effects shown in our study reflect access to somatomotor representations of syllable identity for heard speech in general, and not just due to our use of degraded speech, or due to prior training.

A second concern is that somatomotor responses to speech reflect phonological segmentation and working memory processes associated with active speech perception tasks (Lotto et al. 2009; Sato et al. 2009; McGettigan et al. 2010; Krieger-Redwood et al. 2013). This is consistent with a recent MVPA fMRI study that showed prefrontal and motor representations of speech content during a phoneme identification task (Du et al. 2014). In Du et al. (2014), participants made a 4-alternative forced-choice button press response on every trial to indicate the identity of critical phonemes. Hence, it might have been that motor representations only encoded phoneme identity in order to perform this task. In contrast, in our study, participants only made button press responses when they identified occasional stimulus repetitions. Thus, it seems unlikely that our results can be explained by activity associated with button presses as these were rare (only 7.7% of trials required a response), were modeled separately, and in any case did not directly encode syllable identity.

However, it is harder for us to rule out the possibility that our participants are engaged in a particular listening strategy that requires phonological segmentation, or maintenance of syllables in articulatory working memory. It might be that segmentation or working memory depends on somatomotor representations in a way that natural speech comprehension does not. Indeed, our observation that somatomotor cortex-encoded phoneme identity and syllable structure may be interpreted as consistent with this interpretation. However, as participants detected immediate whole-syllable repetitions, we argue this should have minimized segmentation and working memory demands.

Nonetheless, our task did require that participants actively engaged with the syllables and maintain their attention throughout the scanning run. This may prove to be critical in explaining activation of somatomotor representations, particularly if speech is degraded. A recent fMRI study showed that exogenous distraction reduces recall of degraded but not clear speech and that frontal and motor regions are only activated for degraded speech when it is attended (Wild et al. 2012). Previous studies suggest that the details of the active tasks that participants perform in the scanner are important. Univariate and multivariate imaging studies show differential modulation of neural activity in response to identical stimuli, depending on the feature of the stimulus that participants were instructed to attend to (von Kriegstein et al. 2003; Bonte et al. 2014). Thus, it is unclear whether we would get the same pattern of responses if we had asked participants to attend to a different feature of our stimuli, for example, the speaker rather than the identity of the syllable. We note, however, that Arsenault & Buchsbaum (2015) showed somatomotor representations of articulatory features of heard speech during a speaker gender decision task. Further studies using passive listening or more natural (semantic) listening tasks are necessary, however, for us to delimit the listening situations in which these somatomotor representations become active.

What then, are we to conclude, regarding the role of somatomotor cortex in speech perception? The present results show that the somatomotor responses represent the identity of heard syllables. However, somatomotor cortex is not the only region to encode abstract representations of speech sounds; indeed, our results are consistent with previous findings that temporal cortex also encodes abstract syllable identity (Obleser et al. 2004; Obleser, Boecker, et al. 2006a; Obleser, Scott, et al. 2006b). Our results do suggest that syllable representations in somatomotor regions do not encode speaker identity, suggesting a greater degree of abstraction from the acoustic signal than is seen in lateral temporal regions. Thus, somatomotor responses are unlikely to reflect generic attentional, executive, or decision-making processes (perhaps unlike responses in inferior frontal regions, cf. Binder et al. 2004). While we found evidence for the encoding of syllable identity for both clear and degraded speech, evidence for somatomotor involvement in our work, and in previous imaging (Osnes et al. 2011; Hervais-Adelman et al. 2012; Du et al. 2014) and TMS studies (D'Ausilio et al. 2009; Möttönen and Watkins 2009), is most often observed in challenging listening situations. Our findings are thus consistent with a role for somatomotor regions in supervisory or predictive mechanisms that operate in canonical perception but are most apparent when adapting to degraded or novel speech forms (Adank and Devlin 2010; Erb et al. 2013 for fMRI and Sohoglu et al. (2012) for MEG evidence). Thus, while our findings are to some extent compatible with traditional “motoric” theories of speech perception (Studdert-Kennedy et al. 1970; Liberman and Whalen 2000), we suggest a more nuanced interpretation. We propose that somatomotor representations combine in a top-down fashion (Davis and Johnsrude 2007; Osnes et al. 2011) with auditory representations in order to guide speech perception; perhaps through efferent copy mechanisms similar to those proposed in neural models of speech production (Guenther et al. 2006; Hickok et al. 2011).

## Conclusions

Our findings suggest that links between speech perception and production culminate in abstract representations of heard syllables in somatomotor regions that are also activated during speech production. Activation was increased in adjacent premotor regions during the perception of degraded speech and activity in this region correlated with monitoring accuracy. This is to some extent consistent with analysis-by-synthesis proposed in motor theories of speech perception (Studdert-Kennedy et al. 1970; Liberman and Whalen 2000), and in more recent neural accounts (cf. Davis and Johnsrude 2007; Poeppel and Monahan 2011). However, it is not the case that motor representations can be directly derived from the acoustic signal without several intervening stages of processing. In our data, somatomotor representations of syllable identity are observed at the top of a graded hierarchy mediated by auditory abstraction processes in lateral temporal regions. Thus, we suggest that speech representations in the STG are at an intermediate level between the auditory representations in Heschl's gyrus and the fully abstracted representations observed in somatomotor regions. Understanding the interactive neural computations by which acoustic representations are transformed into more abstract representations is an important goal if we are to understand the neural mechanisms underlying speech perception.

## Funding

This research was funded by the Medical Research Council MC-A060-5PQ80. Funding to pay the Open Access publication charges for this article was provided by the Medical Research Council.
